# Supplementary figures and images for: Comparing eDNA metabarcoding primers for assessing fish communities in a biodiverse estuary
Source: PLoS One. 2022 Jun 17;17(6):e0266720. doi: 10.1371/journal.pone.0266720 (PMC9205523; doi:10.1371/journal.pone.0266720)

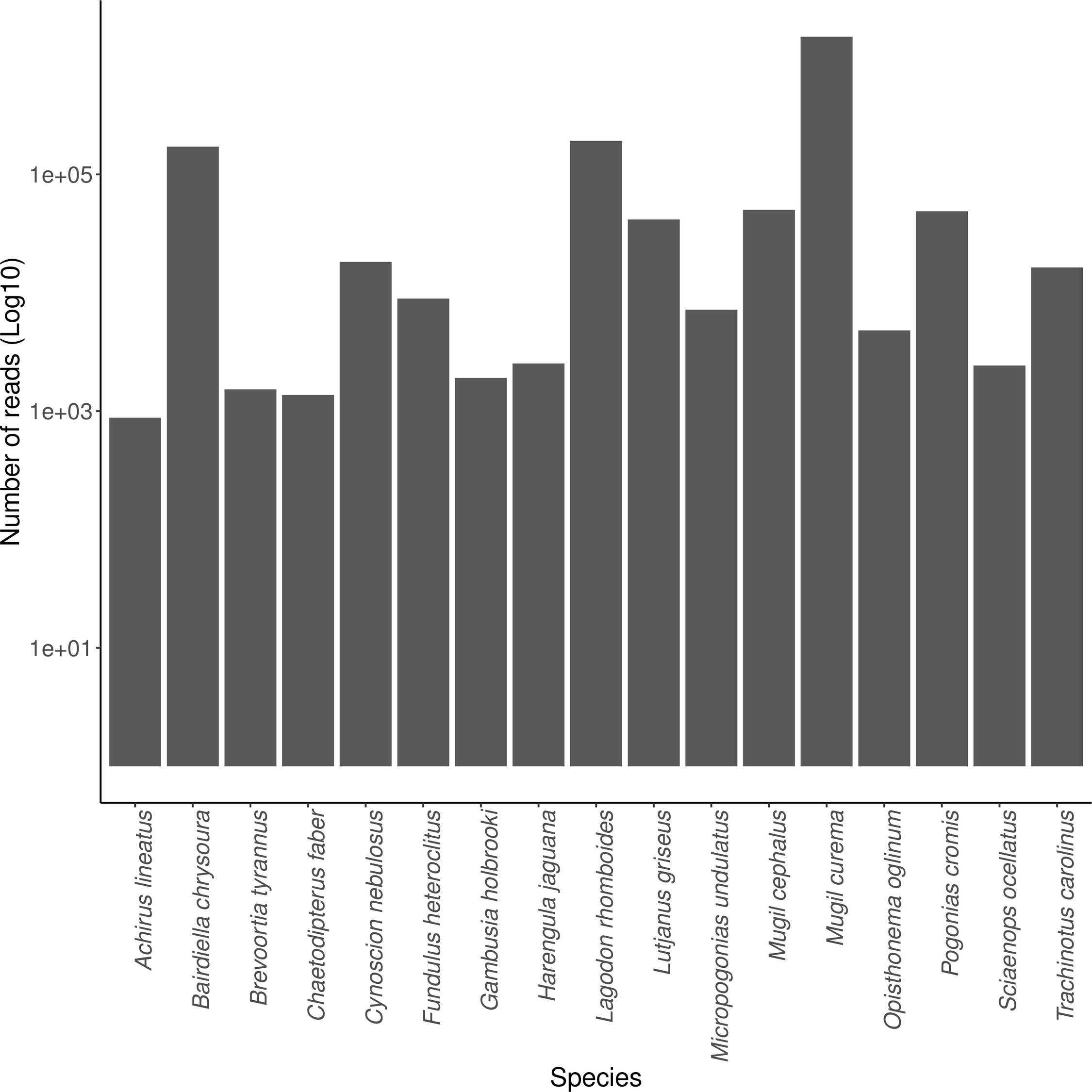

Supplement: S1 Fig — Read numbers are totals across the six sample sites. (TIF) [file pone.0266720.s001.tif]

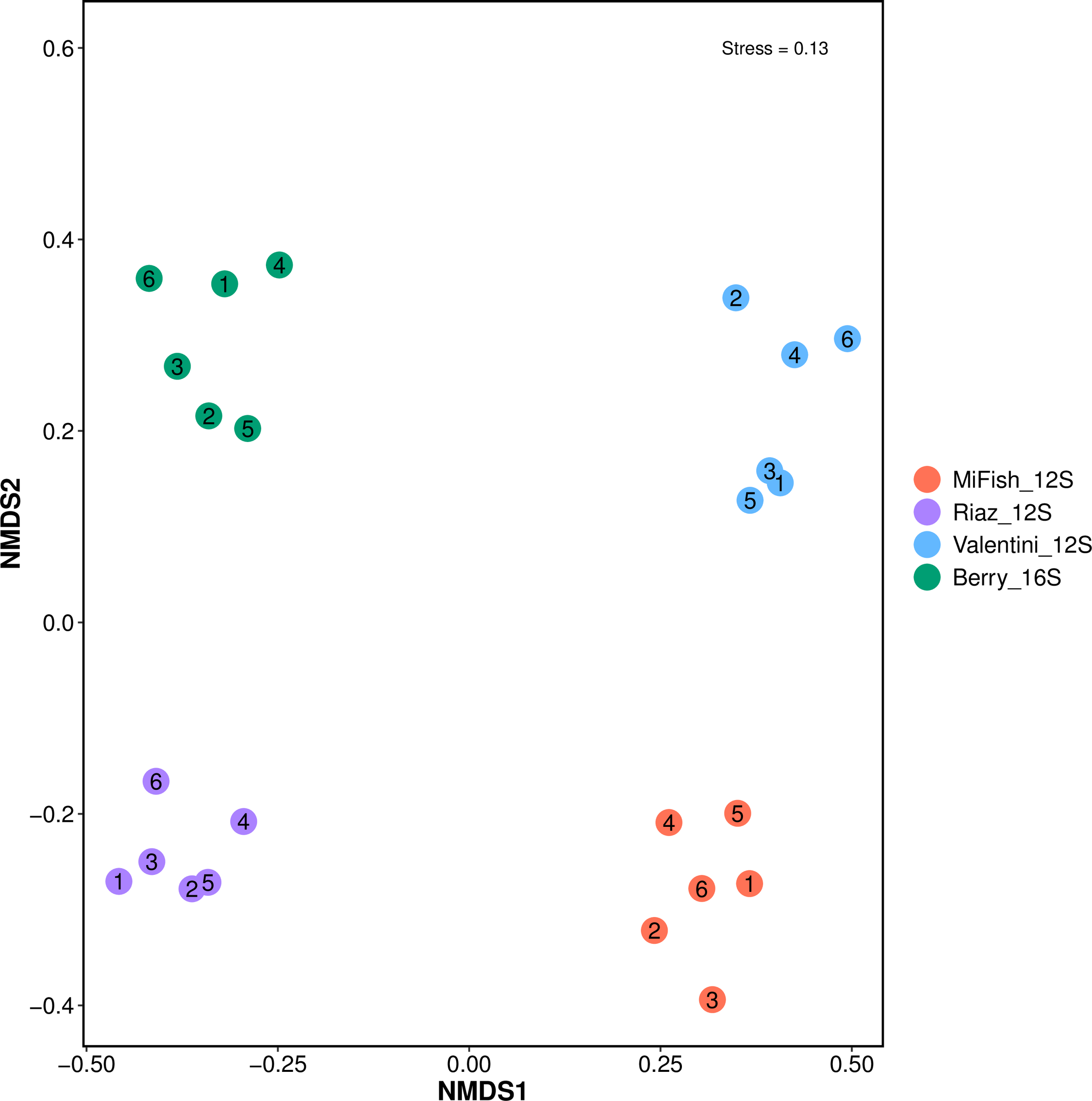

Supplement: S2 Fig — The numbers inside the circles represent sample sites. Metabarcoding data was generated using four primer sets designed to amplify fishes. (TIF) [file pone.0266720.s002.tif]
