# Supplementary material for: Comparing eDNA metabarcoding primers for assessing fish communities in a biodiverse estuary
Source: PLoS One. 2022 Jun 17;17(6):e0266720. doi: 10.1371/journal.pone.0266720 (PMC9205523; doi:10.1371/journal.pone.0266720)
Supplement: S1 Table — Water samples were taken at the surface over shallow water habitat known to have oyster reefs. (DOCX) [file pone.0266720.s003.docx]

**Table S1** Sampling sites used in this study. Two replicates of 500 ml water samples were collected in June 2018 from six locations within the Indian River Lagoon in central Florida. Water samples were taken at the surface over shallow water habitat known to have oyster reefs.

| **Name of site** | **Latitude** | **Longitude** |
| --- | --- | --- |
| Site 1 | 28.969799 | -80.88188 |
| Site 2 | 28.939454 | -80.8669 |
| Site 3 | 28.968022 | -80.879808 |
| Site 4 | 28.944331 | -80.861731 |
| Site 5 | 28.968022 | -80.879808 |
| Site 6 | 28.940124 | -80.867188 |
