# Supplementary material for: Comparing eDNA metabarcoding primers for assessing fish communities in a biodiverse estuary
Source: PLoS One. 2022 Jun 17;17(6):e0266720. doi: 10.1371/journal.pone.0266720 (PMC9205523; doi:10.1371/journal.pone.0266720)
Supplement: S2 Table — Also shown is the percentage of total reads assigned at the species-level (fishes) after quality control. (DOCX) [file pone.0266720.s004.docx]

**Table S2** Total number of fish species detected for each primer set at three different species-level sequence similarity thresholds (99%, 98%, and 97%). Also shown is the percentage of total reads assigned at the species-level (fishes) after quality control.

| **Primer set** | **99% Similarity threshold** | | **98% Similarity threshold** | | **97% Similarity threshold** | |
| --- | --- | --- | --- | --- | --- | --- |
|  | **# Species** | **% Reads assigned to species** | **# Species** | **% Reads assigned to species** | **# Species** | **% Reads assigned to species** |
| MiFish_12S | 34 | 81.39 | 40 | 84.71 | 41 | 84.91 |
| Riaz_12S | 55 | 96.78 | 61 | 97.65 | 61 | 97.65 |
| Valentini_12S | 32 | 86.03 | 37 | 87.43 | 37 | 87.43 |
| Berry_16S | 49 | 97.38 | 51 | 97.86 | 51 | 97.88 |
