# Supplementary material for: Comparing eDNA metabarcoding primers for assessing fish communities in a biodiverse estuary
Source: PLoS One. 2022 Jun 17;17(6):e0266720. doi: 10.1371/journal.pone.0266720 (PMC9205523; doi:10.1371/journal.pone.0266720)
Supplement: S3 Table — Standard deviations were calculated based on the data across all six sample sites. Data for three different species-level sequence similarity thresholds (99%, 98%, and 97%) are shown. (DOCX) [file pone.0266720.s005.docx]

**Table S3** Diversity indices including species richness, evenness, and Shannon’s diversity calculated using the R package BiodiversityR v. 2.12-3 for four metabarcoding primer sets designed to amply fishes. Standard deviations were calculated based on the data across all six sample sites. Data for three different species-level sequence similarity thresholds (99%, 98%, and 97%) are shown.

| **Primer set** | **99% Similarity threshold** | | | **98% Similarity threshold** | | | **97% Similarity threshold** | | |
| --- | --- | --- | --- | --- | --- | --- | --- | --- | --- |
|  | **Species richness** | **Evenness** | **Shannon’s**  **diversity** | **Species**  **richness** | **Evenness** | **Shannon’s**  **diversity** | **Species richness** | **Evenness** | **Shannon’s**  **diversity** |
| MiFish_12S | 22.16 ± 3.13 | 0.134 ± 0.054 | 1.01 ± 0.45 | 27.67 ± 3.38 | 0.133 ± 0.068 | 1.19 ± 0.52 | 28.33 ± 3.27 | 0.131 ± 0.066 | 1.19 ± 0.51 |
| Riaz_12S | 38.0 ± 5.59 | 0.163 ± 0.067 | 1.75 ± 0.47 | 40.5 ± 5.24 | 0.162 ± 0.071 | 1.79 ± 0.51 | 40.66 ± 5.20 | 0.161 ± 0.070 | 1.79 ± 0.51 |
| Valentini_12S | 20.5 ± 4.72 | 0.153 ± 0.054 | 1.09 ± 0.35 | 23.33 ± 5.89 | 0.143 ± 0.041 | 1.15 ± 0.36 | 23.33 ± 5.89 | 0.143 ± 0.041 | 1.15 ± 0.36 |
| Berry_16S | 30.0 ± 6.69 | 0.138 ± 0.064 | 1.30 ± 0.52 | 31.0 ± 6.60 | 0.144 ± 0.079 | 1.33 ± 0.61 | 31.0 ± 6.60 | 0.144 ± 0.079 | 1.33 ± 0.61 |
