# Supplementary material for: Comparing eDNA metabarcoding primers for assessing fish communities in a biodiverse estuary
Source: PLoS One. 2022 Jun 17;17(6):e0266720. doi: 10.1371/journal.pone.0266720 (PMC9205523; doi:10.1371/journal.pone.0266720)
Supplement: S4 Table — Read numbers are totals across all the six sample sites. (DOCX) [file pone.0266720.s006.docx]

**Table S4** The number of reads obtained for each of the 17 fish taxa detected by all four primer sets used in this study. Read numbers are totals across all the six sample sites.

| **Species** | **MiFish_12S** | **Riaz_12S** | **Valentini_12S** | **Berry_16S** |
| --- | --- | --- | --- | --- |
| *Achirus lineatus* | 223 | 236 | 234 | 186 |
| *Bairdiella chrysoura* | 54270 | 45241 | 58322 | 13673 |
| *Brevoortia tyrannus* | 357 | 117 | 319 | 733 |
| *Chaetodipterus faber* | 126 | 44 | 196 | 1001 |
| *Cynoscion nebulosus* | 1434 | 4148 | 5555 | 7071 |
| *Fundulus heteroclitus* | 1646 | 3559 | 3209 | 514 |
| *Gambusia holbrooki* | 372 | 944 | 180 | 408 |
| *Harengula jaguana* | 224 | 707 | 619 | 973 |
| *Lagodon rhomboides* | 14120 | 48105 | 76009 | 53904 |
| *Lutjanus griseus* | 5481 | 9523 | 8636 | 17860 |
| *Micropogonias undulatus* | 1492 | 1701 | 1523 | 2474 |
| *Mugil cephalus* | 16995 | 18182 | 11090 | 3890 |
| *Mugil curema* | 279423 | 334712 | 385558 | 451954 |
| *Opisthonema oglinum* | 326 | 619 | 1306 | 2554 |
| *Pogonias cromis* | 1687 | 40738 | 2329 | 4001 |
| *Sciaenops ocellatus* | 788 | 485 | 261 | 894 |
| *Trachinotus carolinus* | 2841 | 4980 | 537 | 7979 |
| **Total number of reads** | 381805 | 514041 | 555883 | 569976 |
