# Supplementary material for: Comparing eDNA metabarcoding primers for assessing fish communities in a biodiverse estuary
Source: PLoS One. 2022 Jun 17;17(6):e0266720. doi: 10.1371/journal.pone.0266720 (PMC9205523; doi:10.1371/journal.pone.0266720)
Supplement: S5 Table — Total number of reads across all markers and the % of those total reads attributed to each marker is shown. Also listed is the average number of reads per species with the range in parenthesis. (DOCX) [file pone.0266720.s007.docx]

**Table S5** Number of reads that were assigned to fish species at the 99% sequence similarity threshold for each primer set. Total number of reads across all markers and the % of those total reads attributed to each marker is shown. Also listed is the average number of reads per species with the range in parenthesis.

| **Primer set** | **Reads assigned to fish species** | **% of total reads assigned to each marker** | **Average sequencing depth per species** |
| --- | --- | --- | --- |
| MiFish_12S | 404,527 | 16.03 | 11,898 (46 - 249,423) |
| Riaz_12S | 617,235 | 24.46 | 11,430 (8 - 163,690) |
| Valentini_12S | 582,266 | 23.08 | 18,196 (15 - 385,558) |
| Berry_16S | 692,742 | 27.46 | 14,432 (12 - 89,042) |
| **Total** | 2,296,770 |  |  |
