# Supplementary material for: Comparing eDNA metabarcoding primers for assessing fish communities in a biodiverse estuary
Source: PLoS One. 2022 Jun 17;17(6):e0266720. doi: 10.1371/journal.pone.0266720 (PMC9205523; doi:10.1371/journal.pone.0266720)
Supplement: Attachment — Submitted filename: Response to reviewers comment.docx [file pone.0266720.s008.docx]

**Response to reviewer’s comments**

**Reviewer #1**

**Comment:** I have serious concerns on the methods for comparing primers. The usefulness of primers for eDNA metabarcoding should be evaluated the following three aspects, specificity (specific amplification of target taxa), universality (comprehensive amplification of target taxa without bias), and resolution (taxonomic resolution in the amplified region). The manuscript only evaluated primers based on the number of "detected" species.

**Response:** We agree that all three criteria are important and have clarified our goals in the introduction.

“Here we focus on the four best performing primer sets from the freshwater study of Zhang, Zhao (42) and include an additional 18S primer set not previously evaluated (but designed for fishes), and test these in a species rich estuarine system in Florida (the Indian River Lagoon). We compare the efficiency of these primers in terms of 1) specificity (amplification of fishes at the exclusion of other taxa), 2) universality (amplification of a diversity of fish taxa), and 3) taxonomic resolution (ability to resolve to the species level)”.

By this definition we do address specificity. Our target group is fishes. In Table 2 we show the percent of reads that were off target (non-fish). For all primer sets at least 94% of reads assigned to fishes with the exception of the MacDonald primers. We also address universality in terms of the taxonomic levels that were amplified. We can’t directly address the question of sequencing bias with this dataset and therefore we do not attempt to translate read number into relative abundances, but we do include ASV files with read counts and their corresponding sequences. These files have been uploaded to Data Dryad (DOI: https://doi.org/10.5061/dryad.70rxwdbzc). In Table 3 we address the universality issue by highlighting the number of fish taxa (from class to species level) that were amplified, and Fig. 2 shows a Venn diagram of overlapping detections.

**Comment:** The criteria for species identification in this manuscript is ≧99% identity with sequences on the database, but this criteria is not fair. For example, the length of amplified region for Valentini_12S is short, and one base substitution make it genus-level identification according to the authors' criteria.

**Response:** We agree that some primer sets are better candidates for species delimitation. Our goal with this experiment was to determine which primer set allows us to identify the greatest number of fish species). Some gene segments will be better as they are more variable and able to delineate taxa more clearly. This is a function of length but also how the gene evolves and probably of more importance how complete the public databases are. Our goal was not to compare gene regions or consider fragment length, but instead we considered each of these primer sets to be a tool with its assigned task being to identify species in a water sample. Therefore, we evaluated each primer set as a published and established tool (including the public databases) and our goal was to determine which is the best for characterizing fish diversity in our estuarine system.

**Comment:** Also, for MiFish_12S, many previous papers using this primer set adopt the identification threshold at ≧98.5% or lower, and from my inspection of these papers, I believe that the criteria of ≧99% identity is inappropriate. I recommend authors to set an appropriate threshold for each primer set, considering the difference of evolutionary speed among amplified regions. In addition, the accuracy of species determination may vary depending on the richness of the data in the database, so the results may vary greatly depending on the criteria set by the authors.

**Response (L336-344):** Regarding the threshold for the species identification, we used the following criteria for the taxonomic assignment: ≥ 99% similarity for species; 97% for genus; 95% for family; 90% for order; 85% for class; and 80% for phylum following West et al. 2020. The commonly used 97% sequence similarity threshold was proposed for species assignment in 1994 when only few 16S rRNA sequences were available in databases. With increase in the richness of sequences in databases over the years, a variety of sequence similarity threshold (97–100%) have been used in metabarcoding studies using different gene markers. Recent studies have suggested that a 99–100% similarity threshold may be more appropriate for species assignment using 12S (72) and 16S rRNA markers (74). Following these guidelines, we used ≥ 99% similarity cutoff for species assignment.

**Comment:** The above-mentioned specificity, universality, and resolution can be tested via in silico test as well as using the data for field samples. Without such tests, the comparison among primer sets cannot be justified. From above reasons, I think the manuscript should be largely revised before its publication.

**Response (L98-104):** *In-silico* testing of the primer sets used in our study (except McDonald_18S) was performed in Zhang et al. (2020). They found considerable discrepancies between the in-silico and in-vitro results including in the range and diversity of taxa amplified, fish community composition, and the power of each to discriminate species. Since the *in-silico* experiments had already been conducted (and the results were not always in agreement with *in-vitro* results), we went directly to *in-vitro* experiments testing the primers on field samples to evaluate their efficacy for fish biodiversity assessment. The explanation for this comment has been given in lines 98-104.

**Reviewer #2**

**Comment:** The manuscript submitted by Kumar et al. is a simple but well written piece focused on comparing different primer sets for fish eDNA metabarcoding. I would recommend the authors to improve the abstract and methods sections by including more necessary information, especially, regarding the sampling and studied area. For example, it is not very clear were exactly the samples were taken and if brackish or freshwater species are also expected to be recovered from those samples.
The abstract still needs to be modified by clarifying some important information (e.g., target taxonomic group, samples analysed, studied system, etc)

**Response:** We thank the reviewer for pointing out these omissions. We have now included more details about our sample sites, target taxa, etc. in both the abstract and methods sections. Also, a table with GPS coordinates are now including in supplemental materials (Table S1).

**Comment:** L27: Please include the referred ‘efforts’

**Response (L26):** Suggestion has been incorporated**.** Text now reads “The efficiency and outcome of these metabarcoding efforts are dependent upon…”

**Comment:** L29: Include the target taxonomic group

**Response (L29):** The target taxonomic group was fishes. The same has been added in line 29.

**Comment:** L57: remove “a” in “a marine environments”

**Response (L60):** Corrected

**Comment:** L87-89: The authors should better explore this information. The public databases might be more complete for some very few specific taxonomic groups. Therefore, when providing this information, the target group might be included.

**Response (L88-89):** We completely agree with the reviewer’s point of view. The markers used in this study are well represented for fishes in the public databases. The clarification has been given in line 89.

**Comment:** L114: Please include more information for the sampling sites.

**Response:** The details of sample information are now provided in the methods section and Table S1.

**Comment:** L166: How many runs?

**Response (L176-178):** The methods section now reads “Sequencing was conducted using a Nano 300 v2 (2 × 150) Reagent Kit for 2×111 cycles and a Nano 500 v2 (2 × 250) Reagent Kit for 2×251 cycles, based on amplicon size.”

**Comment:** L288: Insert a space between ‘and’’16S’

**Response:** Corrected

**Comment:** It is also important to note and discuss the lack of appropriate reference sequences. Despite being known that the combination of multiple primers targeting distinct gene is expected to increase taxonomic resolution and consequently, the number of species detected, the lack of references remains as a hindrance.

**Response (L325-344):** We completely agree that the effectiveness of eDNA metabarcoding studies are highly dependent on the completeness and accuracy of the relevant reference databases. We have discussed this issue in lines 325-344.

**Reviewer #3:**

**Comment:** The authors performed a comparative study, aimed at evaluating the performances of different primer sets specifically designed to target fish species, in a highly biodiverse system: the Indian River Lagoon in Florida. They tested three 12S and one 16S rRNA primer sets, and a 18S primer set designed for freshwater fish that did not produce useful results. I found the manuscript clear, and I think its findings could be helpful for choosing the best primer sets for studying fish diversity using metabarcoding. However, I think that authors could make a bigger effort to present the differences among the tested primer sets in a more appropriate way. The figures also need to be supported by more precise legends.
**Response:** We thanks the reviewer for their thoughtful comments. We have improved the manuscript by adding more detailed information on sampling and plotting nMDS using both read abundance data as well as presence/absence data. The figure legends have been elaborated to include all the necessary information.

**Comment:** Line 53-54: Here you cite some works aimed at describing environmental DNA metabarcoding in general. That is fine, except maybe for the paper of Kumar, Eble and Gaither (2020), that I find a little out of topic. I think that you should rather cite some empirical studies supporting your sentence. For example, the recent work of Aglieri et al. (2020) compared simultaneously three traditional sampling methods with environmental DNA metabarcoding, finding complementarity among the methods.

**Response:** We have removed the review papers here and added a few empirical studies that better support this statement including Aglieri et al. (2020).

**Comment:** Line 98: duplicated “in”

**Response:** Corrected

**Comment:** Line 209: Except for species richness, the diversity indexes you used need abundance data. You used to read numbers as a proxy for abundance, but I am not so much a supporter of this approach. PCR biases can alter the proportion between the amount of initial DNA template and the final sequences yield. This is even more worthy of consideration when two PCRs are used to build the sequencing libraries, as you did. Moreover, the amount of genetic material in the water is not necessarily related to the abundance of individuals: different species have different dimensions, different shedding, etc. Several authors have made the attempt of relating sequence abundance to individual abundance, but the relation is very often poor. That is a current limit of metabarcoding, and at least you should explain that. Please, add a few lines to address this point.
**Response (L345-353):** We largely agree with the reviewer here. Even though several studies have shown positive correlations between abundance estimates from eDNA metabarcoding data (reviewed by Rourke et al. 2020), the correlation is often weak. We have address this comment in lines 345-353.

**Comment:** Line 215: As in the previous comment. Bray-Curtis uses abundances. I would like to see also something made using presence/absence data, at least to verify the differences with the abundance related indexes you used. For example, you could show the same nMDS, but made with the Sørensen index. Please, show something more.

**Response:** As per the suggestion, we plotted the nMDS using abundance data as well as presence/absence data. Both the data set showed clear separation among primer sets. The nMDS plot using presence/absence data has been presented as a supplementary figure S2.

**Comment:** Line 216-217 and Figure 3: The nMDS plot shows six points for each primer set. I don’t get what they represent, since you sampled three sites taking four replicates for each one. The plot should show three points, in the case you pooled the replicates for each site, or 12 points if you were showing the diversity for each replicate. Please explain better what the points represent.
**Response (L120-123):** The six points in the nMDS plot represent the six sampling sites used in this study. From each site two field replicate water samples (500 ml per sample) were collected. The details of the sampling information has been provided in Materials and Methods section in lines 120-123 and Table S1.

**Comment:** Line 222: Since you decided to consider the abundance of reads to calculate diversity indexes, why don’t you also show the number of reads of the taxa in common among the different primer sets? This could be indicative of the amplification efficiency of each primer set for those taxa. This is just a suggestion, but I would like to understand more.

**Response (L285-289):** The number of reads of the taxa in common among the different primer sets have been provided in lines 285-289. The number of reads for the taxa in common among the Riaz_12S, Valentini_12S, and Berry_16S primer sets were comparable and 514,041, 555,883, and 569,976 reads were assigned to each primer set, respectively. The MiFish_12S has the lowest number of reads (381,805) assigned to species in common.

**Comment:** Line 232: Please, could you provide also the average number of reads for sample?

**Response (L245-248):** The average number of reads per sample for each marker set has been included in lines 245-248.

**Comment:** Line 261-262: Low evenness means big differences in reads number among taxa. This is quite common in metabarcoding studies, since several variables can influence the yield, resulting in high variability. Anyway, how different these yields are for each taxon? Please, could you provide something more? For instance, a plot with all the reads of each taxon ordered from the smaller to the higher. Alternatives to my suggestion are also welcome.

**Response:** As per the suggestion we plotted a graph of 17 common species detected across all four primer sets, with reads ordered from the smaller to the higher. The figure has been presented as a supplementary figure S1.
