# Supplementary material for: Comparing eDNA metabarcoding primers for assessing fish communities in a biodiverse estuary
Source: PLoS One. 2022 Jun 17;17(6):e0266720. doi: 10.1371/journal.pone.0266720 (PMC9205523; doi:10.1371/journal.pone.0266720)
Supplement: Attachment — Submitted filename: Response to reviewers comments_F.docx [file pone.0266720.s009.docx]

**Response to reviewer’s comments**

**Reviewer #1**:

**Comment:** The authors kept ≥ 99% similarity cutoff for species assignment regardless of the previous review comments. The threshold should be set for each primer set because the evolutionary rate varies even within the same gene. From my experience for example, The MiFish region clearly has a fast evolutionary rate, and one underestimates the species detection when a 99% threshold is adopted. The threshold value should be set carefully because it has a significant impact on the validity of primer selection.

**Response:** We agree. As per the suggestion of the reviewer, we used three different similarity cutoffs (97%, 98%, and 99%) to see if the change in threshold criteria influenced species detection. An increase in the total number of detected species was observed when we used 98% cutoff as compared to 99% similarity threshold (Table S2; below). However, a comparison of 98% to 97% similarity threshold did not lead to a change (except one species for MiFish). Similar to taxonomic assignment, different cutoffs for the primers did not change the overall diversity indices (Table S3). Since the overall result did not change, we decided to use 99% similarity threshold to avoid any misidentification of species.

**Table S2** Fish species detected for each primer set at each of three different similarity thresholds (99%, 98%, and 97%). Also shown is the percentage of total reads assigned at the species-level (fishes) after quality control.

| **Primer set** | **99% Similarity threshold** | | **98% Similarity threshold** | | **97% Similarity threshold** | |
| --- | --- | --- | --- | --- | --- | --- |
|  | **# Species** | **% Reads assigned to species** | **# Species** | **% Reads assigned to species** | **# Species** | **% Reads assigned to species** |
| MiFish_12S | 34 | 81.39 | 40 | 84.71 | 41 | 84.91 |
| Riaz_12S | 54 | 96.78 | 61 | 97.65 | 61 | 97.65 |
| Valentini_12S | 32 | 86.03 | 37 | 87.43 | 37 | 87.43 |
| Berry_16S | 48 | 97.38 | 51 | 97.86 | 51 | 97.88 |

**We’ve added this text to section 3.2**

“While adjusting the sequence similarity threshold did change the number of species detected for each primer set the overall pattern did not change. Regardless of which similarity threshold was applied the Riaz_12S primer set resulted in the most species detected with Berry-16S ranking second (Table S2). There were no notable changes in taxa detected when comparing the 97% and 98% thresholds but there was an increase in the number of species resolved if we applied the 98% threshold compared to the more conservative 99% threshold. Most notably, when we compared the best performing Riaz_12S using the 99% cutoff with the worst performing primers sets using a 97% cut off the latter still resulted in high number of specie detections (Table S2). Similar patterns were found for diversity indices (Table S3). As a result, we have opted to apply a single conservative similarity threshold of 99% for species-level designations and report these throughout.”

**Reviewer #4:** In my opinion, the paper is mainly sound. However, the authors clarified the candidate primers were chosen according to Zhang (2021). Zhang (2021) is based on freshwater fish and suggested that the primer choice cannot be solely based on in silico evaluation. As the author said by themselves, primer selection experiments should do both in silico and vitro. So I think that's not enough to justify the choice of these candidate primers. The author should clarify this point in the discussion because marine samples are very different from freshwater samples. Otherwise, this study doesn't have a customer database to compare the outcome species, but it's fine as it's fair to all primers. They only use the inventory list alone is not enough to say that they did an excellent species resolution survey. In short, the authors show how they selected primers for their study in an estuarine environment. It's a good complement to the eDNA study. As a methodology study, this study is not rigorous enough to reflect the value of the title. The authors already account for the shortcomings of their experimental design in the revised edition. I suggest authors should also clarify the limitations of candidate primer selection.

**Response:** The choice of primer candidates has been further explained in the discussion section. Zhang et al. (2020) performed *in silico* experiments on a number of primer sets including 4 of the 5 selected here. For *in vitro* experiments they tested these same primer sets in a freshwater system. The title of the manuscript has been changed to reflect our study.

Our Discussion now reads

“Despite the growing interest in the use of eDNA to assess fish communities, there has been a surprising lack of studies that have directly compared the efficacy of metabarcoding primer sets in marine and estuarine systems. Because the performance of eDNA metabarcoding primers will vary depending on the study system (freshwater, marine, or estuarine) and taxonomic composition, there is no guarantee that a primer set that performs well in a freshwater system will do so in marine or estuarine systems. For this reason, we tested the four best performing metabarcoding primers from the freshwater study of Zhang, Zaho (42), to determine their effectiveness in a biodiverse estuarine system.”

**Reviewer #5:**

**Comment:** I generally have serious concerns regarding the idea of comparing primers that have such a different amplicon length and especially I agree with reviewer #1 in the criticism about the criteria for species identification, using the same threshold for example for Valentini 12S that is 63 bp long and for MiFish 12S, which is nearly three times that length.

**Response**: The reviewers point out the important point that the primers used in this study produce different amplicon lengths which may influence species detection. It is also important to take into consideration that when a lab picks up a new protocol, they are looking for clear guidance concerning thresholds and cuts-off but in general this is lacking in the literature. As a result, general rules of thumb are typically applied such as 99% for species level designations. With this in mind we now present some analyses using three different similarity cutoffs (97%, 98%, and 99%) to determine if changes in threshold criteria influence the total number of species detected. Overall interpretation of the results did not change, and our results section (Section 3.2) now reads:

“While adjusting the sequence similarity threshold did change the number of species detected for each primer set the overall pattern did not change. Regardless of which similarity threshold was applied the Riaz_12S primer set resulted in the most species detected with Berry-16S ranking second (Table S2). There were no notable changes in taxa detected when comparing the 97% and 98% thresholds but there was an increase in the number of species resolved if we applied the 98% threshold compared to the more conservative 99% threshold. Most notably, when we compared the best performing Riaz_12S using the 99% cutoff with the worst performing primers sets using a 97% cut off the latter still resulted in high number of specie detections (Table S2). Similar patterns were found for diversity indices (Table S3). As a result, we have opted to apply a single conservative similarity threshold of 99% for species-level designations and report these throughout.”

Furthermore, our discussion now reads

“Because the rate of evolution varies across genes and gene regions applying a single set of taxonomic thresholds (i.e., 99% for species; 97% for genus; 95% for family; 90% for order; 85% for class; and 80% for phylum; as applied here) can be misleading. However, our analyses of the sequencing results from the four primer sets at three different species-level similarity thresholds (99%, 98%, and 97%) did not change our interpretation of the data. The Riaz_12S primer set either did as well or outperformed the others in terms of specificity (99.99% of reads assigned to fishes; Table 2), universality (greatest number of taxa amplified; Table S2), and taxonomic resolution (96.78% of reads assigned to the species-level at the 99% threshold) regardless of which cutoff value was applied. Most notably, this was true even when comparing Riaz_12S using the conservative 99% cutoff against all other primers sets using a 97% cut off (Table S2). Similar patterns were found for diversity indices (Table S3). It’s important to note that the Berry_16S primer set also performed well in our experiments.”

**Comment:** the authors used reads numbers as a proxy for abundance data and compared the results for the four primer sets. A lot of factors can affect the proportion between the initial amount of template DNA and the final numbers of sequence reads. This is even more an issue considering the different lengths of the fragment amplified and the fact that you used two PCRs to build sequencing libraries. I would suggest to use the proportions instead of the raw number of reads or at least transform the number of reads (e.g. forth square or logarithm) to make results more comparable.

**Response:** We thank the reviewer for this suggestion. We have log transformed the sequence reads of the 17 common species among all the four primer sets used in this study and a figure presenting these data can now be found as Figure S2.

Our text now reads:

“Based on the ANOSIM analyses, species assemblages differed significantly across primer sets (*R* = 0.4011, *P* < 0.001); a finding that was supported by the NMDS plots which showed clear separation among marker sets when either the read abundance data (Figure 3) or presence/absence data (Figure S2) were analyzed.”

**Comment:** for a better comparison of the efficiency of the four different primer sets I would suggest the author to take into account not only the number of generated reads per marker but also their relative sequencing depth, especially for those sequences that were assigned to fishes.

**Response:** Percent of total reads per marker and average sequencing depth per species were calculated and data is now presented in Table S5. Our results were further corroborated as MiFish_12S resulted in the number of reads suggesting lower PCR efficiency for this primer set in our experiments.

**Comment:** The authors state that they performed two replicates in each of the six sampling sites but then in the nMDS plot I can see only 6 points. Were the replicates pooled? How? I think this should be specified, as it will also affect species richness values within samples.

**Response:** The sequence reads of replicates from the same sites were pooled to plot NMDS. This has been clarified in the text in line 230..

**Comment:** I agree with reviewer #2 and I think you should add more details on the sampling sites. I would suggest to add a map showing the geographic coordinates of each sampling site or at least add more details in Table S1. Latitude and longitude records should obviously include N/S and W/E annotations respectively.

**Response:** The details of our study sites has been added to Table S1 with GPS coordinates in decimal degrees (DD) which is the preferred format for GEOME where we archive our metadata. DD are not typically reported with N/S or W/E annotations.
