# Supplementary material for: Comparing eDNA metabarcoding primers for assessing fish communities in a biodiverse estuary
Source: PLoS One. 2022 Jun 17;17(6):e0266720. doi: 10.1371/journal.pone.0266720 (PMC9205523; doi:10.1371/journal.pone.0266720)
Supplement: Attachment — Submitted filename: Response to reviewer_F.docx [file pone.0266720.s010.docx]

**Response to Editor**

**Comment:** The study compared the primer pairs but did not make an attempt to describe the differences in biological resolution. Do all primer pairs have the same power to capture differences between locations (or any other biological variable of interest, habitats, seasons, depth etc.)? It is not described in the methods (or I missed it) but do the 6 sites differ biologically?
This can be tackled easily; can you add location numbers to NMDS graph. Also, can you test for differences between sites?
Please bring this point also up in the discussion.

**Response:** Thank you for the positive feedback on our manuscript. Our sample sites don’t represent different habitats but instead are over a similar bottom type and are in close proximity. Therefore, we consider them to be replicates. However, that still begs the question of whether they are different. So, as you suggested, we performed the ANOVA to determine if the sites differ. Results showed no significant differences among the sites (p = 0.33), suggesting that the sampling sites are biologically similar. In the NMDS plot (figure 3), we have added the location numbers and clarified in the legend of figure. All these points have been clarified in the methods/results and mentioned in the discussion section.

**Minor points.**

Line 64
“breadth of which can vary from”
**Response:** Corrected

Line 98.
References to the Zhang paper, no need to include the first name, “Zhang et al” is better
“In a recent study, Zhang et al. (42)”
**Response:** Done

Line 209. Extra word in citation?
“following West, Stat (35).”
**Response:** Citation has been corrected.

Line 230-31. Another reference issue”
“An analysis of similarity (ANOSIM; ANOSIM; 55)”
**Response:** Duplicate ANOSIM has been deleted.

Line 272. Add “predictably”
“but there was predictably an increase in the number”
**Response:** “Predictably” has been added in the sentence.

Line 321.
Is there any guarantee that the primers found here will be best in other marine or estuarine systems?

**Response:** There is no guarantee that performance of primers in this study will be best in other marine or estuarine system as effectiveness of primers differ depending on the geographic regions and different ecosystems. The same has been discussed in the Conclusions and Recommendations section.

Line 350. Ref issues again, sort through entire manuscript and fix.
“MacDonald, Young (44)”

**Response:** Corrected

Line 365 Please rephrase.
“However, careful testing is still needed.”
**Response:** The section now reads “However, if elasmobranchs are the primary target group, it would be prudent to test primers using DNA extractions from species expected in the study area. Furthermore, there are a number of published primers that have been designed specifically for elasmobranchs that are worth exploring”
